# Supplementary material for: Overexpression of Ssd1 and calorie restriction extend yeast replicative lifespan by preventing deleterious age-dependent iron uptake
Source: eLife. 2026 Jan 12;14:RP108892. doi: 10.7554/eLife.108892 (PMC12795504; doi:10.7554/eLife.108892)
Supplement: Supplementary file 1. [file elife-108892-supp1.docx]

**Supplemental Table 1:** **Yeast strains used in this study**

| IGY138 | MATα *his3Δ* *leu2Δ ura3Δ ssd1*::NatMX |
| --- | --- |
| IGY081 | MATα *his3Δ* *leu2Δ ura3Δ SSD1*-GFP (KanMX6) |
| IGY086 | MATα *his3Δ* *leu2Δ ura3Δ* P_GPD1_-*SSD1*-GFP (KanMX6) |
| IGY536 | MATα *his3Δ* *leu2Δ ura3Δ SSD1*-mCherry (*HIS3*) |
| IGY540 | MATα *his3Δ* *leu2Δ ura3Δ* P_GPD1_-*SSD1*-mCherry (*HIS3*) |
| IGY106 | MATα *his3Δ* *leu2Δ ura3Δ SSD1*-GFP (KanMX6) *PAB1*-mCherry (*HIS3*) |
| IGY115 | MATα *his3Δ* *leu2Δ ura3Δ* P_GPD_-1*SSD1*-GFP (KanMX6) *PAB1*-mCherry (*HIS3*) |
| IGY107 | MATα *his3Δ* *leu2Δ ura3Δ SSD1*-GFP (KanMX6) *EDC3*-mCherry (*HIS3*) |
| IGY110 | MATα *his3Δ* *leu2Δ ura3Δ* P_GPD1_-*SSD1*-GFP (KanMX6) *EDC3*-mCherry (*HIS3*) |
| IGY117 | MATα *his3Δ* *leu2Δ ura3Δ SSD1*-GFP (KanMX6) *HSP104*-mCherry (*HIS3*) |
| IGY111 | MATα *his3Δ* *leu2Δ ura3Δ* P_GPD1_-*SSD1*-GFP (KanMX6) *HSP104*-mCherry (*HIS3*) |
| IGY544 | MATα *his3Δ* *leu2Δ ura3Δ SSD1*-mCherry (HIS3) URA3-P_GPD1_-GFP_*AFT1* |
| IGY545 | MATα *his3Δ* *leu2Δ ura3Δ* P_GPD1_-*SSD1*-GFP-mCherry (*HIS3*) *URA3*-P_GPD1_-GFP_*AFT1* |
| IGY521 | MATα *his3Δ* *leu2Δ ura3Δ SSD1*-GFP (KanMX6) *ARN1*-mCherry (*HIS3*) |
| IGY522 | MATα *his3Δ* *leu2Δ ura3Δ* P_GPD1_-*SSD1*-GFP (KanMX6) *ARN1*-mCherry (*HIS3*) |
| IGY529 | MATα *his3Δ* *leu2Δ ura3Δ SSD1*-GFP (KanMX6) *FIT2*-mCherry (*HIS3*) |
| IGY530 | MATα *his3Δ* *leu2Δ ura3Δ* P_GPD1_-*SSD1*-GFP (KanMX6) *FIT2*-mCherry (*HIS3*) |
| IGY516 | MATα *his3Δ* *leu2Δ ura3Δ SSD1*-GFP (KanMX6) *aft1*::*HIS3* |
| IGY524 | MATα *his3Δhis3Δ* *leu2Δ ura3Δ* P_GPD1_-*SSD1*-GFP (KanMX6) *aft1*::*HIS3* |
